# Extracellular vesicle-mediated regulation of macrophage polarization in bacterial infections

**DOI:** 10.3389/fmicb.2022.1039040

**Published:** 2022-12-22

**Authors:** Mingjuan Qu, Hongwei Zhu, Xingxiao Zhang

**Affiliations:** ^1^School of Life Sciences, Ludong University, Yantai, China; ^2^Yantai Key Laboratory of Animal Pathogenetic Microbiology and Immunology, Yantai, China; ^3^Shandong Provincial Key Laboratory of Quality Safety Monitoring and Risk Assessment for Animal Products, Jinan, China; ^4^Shandong Breeding Environmental Control Engineering Laboratory, Yantai, China

**Keywords:** extracellular vesicles, exosome, macrophage polarization, membrane vesicles, bacterial infectious diseases

## Abstract

Extracellular vesicles (EVs) are nanoscale membrane-enveloped vesicles secreted by prokaryotic and eukaryotic cells, which are commonly defined as membrane vesicles (MVs) and exosomes, respectively. They play critical roles in the bacteria–bacteria and bacteria–host interactions. In infectious diseases caused by bacteria, as the first line of defense against pathogens, the macrophage polarization mode commonly determines the success or failure of the host's response to pathogen aggression. M1-type macrophages secrete pro-inflammatory factors that support microbicidal activity, while alternative M2-type macrophages secrete anti-inflammatory factors that perform an antimicrobial immune response but partially allow pathogens to replicate and survive intracellularly. Membrane vesicles (MVs) released from bacteria as a distinctive secretion system can carry various components, including bacterial effectors, nucleic acids, or lipids to modulate macrophage polarization in host–pathogen interaction. Similar to MVs, bacteria-infected macrophages can secrete exosomes containing a variety of components to manipulate the phenotypic polarization of “bystander” macrophages nearby or long distance to differentiate into type M1 or M2 to regulate the course of inflammation. Exosomes can also repair tissue damage associated with the infection by upregulating the levels of anti-inflammatory factors, downregulating the pro-inflammatory factors, and regulating cellular biological behaviors. The study of the mechanisms by which EVs modulate macrophage polarization has opened new frontiers in delineating the molecular machinery involved in bacterial pathogenesis and challenges in providing new strategies for diagnosis and therapy.

## Introduction

Macrophages are heterogeneous cells, distributed in various tissues and organs, and participate in innate immunity, antigen presentation, and anti-infectious immune regulation (Murray, 2017). During the inflammatory responses evoked by microorganisms, they can be activated and differentiated into two subtypes with distinct phenotypes and functions under the induction of microenvironment (Essandoh et al., 2016). M1 macrophages, known as an inflammatory subtype, have a strong bactericidal function by releasing pro-inflammatory cytokines, while excessive activation can also aggravate inflammatory responses, lead to tissue injury, and contribute to pathogenesis. M2 macrophages, also known as an anti-inflammatory subtype, can promote tissue repair and immune regulation by secreting anti-inflammatory or immunomodulatory cytokines. In the development of inflammation, these two subtypes are in a dynamic equilibrium and can convert into each other under certain conditions (Smith et al., 2016). There are many factors leading to different polarization of macrophages, including physiological and pathological environments, microbes and their products, cytokines, and activated lymphocytes (Lawrence and Natoli, 2011). In recent years, converging researchers have reported that the potential biological function of extracellular vesicles (EVs) released from cells *in vivo* and *in vitro* exerts essential roles in macrophage polarization to maintain physiological homeostasis (Schorey et al., 2015; Jurkoshek et al., 2016; Jones et al., 2018; Furuyama and Sircili, 2021).

Extracellular vesicles are small membranous vesicles secreted to the extracellular environment by various types of eukaryotic cells including immune and non-immune cells (Denzer et al., 2000). They can be used as carriers for information exchange and transmission between cells, modulate cellular activities, and reprogram the phenotype in recipient cells (Schorey et al., 2015). EVs contain abundant biomolecules, which can modulate the balance of M1/M2 macrophages polarization through different pathways in inflammatory diseases such as tuberculosis (TB), Crohn's disease (CD), sepsis, and pneumonia (Saadatpour et al., 2016). Bacteria can also secrete EVs, which are known as bacterial membrane vesicles (BMVs), containing distinct components including toxins, virulence factors, nucleic acids, and other molecules that promote survival in the host and modulate the host immune response positively or negatively (Jurkoshek et al., 2016; Owen et al., 2016; Jones et al., 2018). Some EVs stimulate innate and adaptive immune responses and promote major histocompatibility complex (MHC) class II antigen presentation of dendritic cells to eliminate bacterial infection (Jurkoshek et al., 2016; Cheng and Schorey, 2019; Lee et al., 2020). In contrast, other vesicles can inhibit the activation of naïve macrophages and suppress the expression of MHC-II in mouse bone marrow-derived macrophages (BMMs) or activate M2 macrophages, which play an anti-inflammatory role and contribute to bacteria evading from surveillance of the immune system (Singh et al., 2011; Ti et al., 2015; Furuyama and Sircili, 2021). Therefore, the regulation of macrophage polarization by EVs from varying sources is a double-edged sword. Revealing the composition of EVs in the course of bacterial infection and the mechanism of how these vesicles promote macrophage polarization can clearly help understand the development of inflammation progress and provide a new strategy for preventing microbe-induced inflammation aggravation. In this review, we systematically summarize the current state of knowledge about the roles of EVs secreted from both bacteria and bacteria-infected cells in modulating macrophage polarization in various inflammatory diseases caused by common bacteria and enumerate the possible mechanisms of how these EVs activated macrophages differentiation, which can be used as a diagnostic biomarker or target for the prevention and treatment of bacterial infection.

## Mechanism of macrophage polarization

Macrophages are plastic and heterogeneous cells due to different mechanisms governing their differentiation. Tissue distribution with different microenvironments, such as intestines, alveolar space, or adipose tissue, may also constrain the functional properties of macrophages (Benoit et al., 2008). The presence or absence of microbial infection *in vivo* or *in vitro* is also necessary for macrophage polarization. Although the use of the terms M1 and M2 remains controversial due to the lack of a tightly defined criterion for scoring the increasing number of activated macrophage subtypes, efforts to define polarization are ongoing, and polarized macrophages have been a partial consensus to be classified into two groups: M1 and M2 macrophages (Murray et al., 2014; Murray, 2017). Macrophages typically exist between these two groups, as the polarization process is dynamic and cells often display characteristics of both states simultaneously. Summarizing the key experimental findings on macrophage polarization in bacterial infection will attempt to define some of the major questions in this field.

### M1 activation in bacterial infection

Macrophage polarization can occur at any point in an inflammatory process and can also be typically evoked *in vitro* by treating cells with lipopolysaccharide (LPS) and/or interferon-γ (IFN-γ). Numerous studies suggest that macrophages exposed to pathogens respond with common transcriptional activation programs. In the early course of infection, as the first line of defense against pathogens, macrophages can recognize and respond rapidly to invading microbes by the expression of pattern recognition receptors (PRRs) such as toll-like receptor 2 (TLR2) or TLR4 and nucleotide-binding oligomerization domain (NOD)-like receptors (NLRs), which show increased susceptibility to gram-negative and gram-positive bacterial infections, respectively (Elson et al., 2007). Then macrophages are activated to secrete inflammation-related cytokines such as interleukin-6 (IL-6), IL-1β, tumor necrosis factor-α (TNF-α), and chemokines such as C–C motif chemokine 2 (CCL2) and CCL5 to recruit more macrophages and neutrophils to eliminate invasive microorganisms (Murray, 2017). CD86 and CD80 are also expressed in M1-polarized macrophages as surface markers, and iNOS (also called NO synthase 2, NOS2) is upregulated to synthesize more NO, which is an important messenger and effector molecule in the defense system of macrophages (Jungi et al., 1997; Barley et al., 2022). These factors potently contribute to the establishment of a pro-inflammatory activation state, which is commonly referred to as classical M1 macrophage activation (Bhatnagar et al., 2007; Hui et al., 2018). The activation can be further induced by cytokines such as IFN-γ released from activated T helper 1 (Th1) cells. Nuclear factor-kappa B (NF-κB) and signal transducer and activator of transcription 1 (STAT1) impact on M1 polarization are involved in the infection progress (Murray, 2017). It is becoming clear that M1-polarized macrophages are associated with the control of acute infections such as active tuberculosis and gastroenteritis in the early phases of healing and high antigen presentation, while an excessive or prolonged M1 activation is deleterious for the host (Mège et al., 2011; Owen et al., 2016; Liu et al., 2021).

### M2 activation in bacterial infection

The M2-polarized macrophages are known as an anti-inflammatory phenotype with a low phagocytic and bacterial killing ability, which are generally prominent in the later course of bacterial infections to prevent tissue damage (Atri et al., 2018). M2 macrophages are characterized by functional expression of anti-inflammatory cytokine mediators such as IL-10, transforming growth factor-beta (TGF-β), and Arginase-1 (Arg-1), as well as alternative activation markers CD206, CD163, mannose receptor, IL-4R, and chemokines that will extenuate inflammatory reactions and promote the wound healing process and tissue repair (Arnold et al., 2007; Paynich et al., 2017). STAT6, STAT3, peroxisome proliferator-activated receptor delta (PPARδ), or PPZRγ impact is involved in the progress of M2 polarization, respectively, which is similar in M2 program evoked by IL-4 and/or IL-13 treatment *in vitro* (Murray, 2017; Stapels et al., 2018; Panagi et al., 2020). The specific M2 phenotype of macrophages with low bactericidal ability is also thought to be beneficial to bacterial pathogens' immune escape for survival (Kyrova et al., 2012; Wang, Y et al., 2021). Though M2-polarized macrophages are prominent in the reparative phase of inflammation, they are also found in connection with parasitic and chronic bacterial infections such as sepsis and Buruli ulcer (Kiszewski et al., 2006; Wang, X et al., 2021). The balance of switching between the M1 and M2 polarization states is necessary to allow the beneficial processes of inflammation, resolution, and repair.

## Biogenesis and composition of EVs

### Membrane vesicles in bacterial infections

During the bacterial infection, nanosized EVs are released to extracellular space by both the host and bacteria (Pfeifhofer-Obermair et al., 2016; Escudé Martinez de Castilla et al., 2021; Tian et al., 2022). Bacterial vesicles are broadly defined as MVs with a size range from 20 to 300 nm (Palacios et al., 2021), which are enriched for LPS, phospholipids, peptidoglycan (PG), periplasmic and cytoplasmic proteins, and nucleic acids, respectively (Schwechheimer and Kuehn, 2015; Jan, 2017; Furuyama and Sircili, 2021). Proteomics analysis implied that MVs from different bacterial sources were diverse in composition (Williams et al., 2007; Choi et al., 2011; Lee et al., 2015), indicating that various bacterial infections caused different inflammatory responses. The selectivity of MV cargo seems a deliberate process, rather than a random event, though the biogenesis mechanism of cargo selection is still ambiguous (Liu et al., 2022). The biogenesis of MVs is supposed to be regulated by multiple elements, including genetic background and growth conditions such as temperature, stress factors, oxidation state, iron, vasculogenesis, and immune response regulators (VirRs) (Jan, 2017; Furuyama and Sircili, 2021; Palacios et al., 2021). A high-throughput screen of a whole-genome knockout library of *Escherichia coli* (*E. coli*) identified nearly 150 genes that affect vesicle biogenesis (Kulp et al., 2015). The vesicles of some bacteria have bacteriolytic enzymes capable of distinguishing between self and non-self microbes and killing other bacteria that surround them (Yaron et al., 2000; Caruana and Walper, 2020). Meanwhile, bacterial MVs during infection could be recognized by immune cells through cell surface TLRs (Jurkoshek et al., 2016). For instance, TLR2, TLR4, and TLR5 located at the host cell membrane are reported to recognize bacterial lipoproteins and flagellins, while TLR7, TLR8, and TLR9 located at the endosomal membranes can bind MV-associated nucleic acids, respectively (Jurkoshek et al., 2016; Liu et al., 2022). The pathways of MVs from different bacteria entry into host cells include endocytosis (e.g., clathrin-mediated, caveolin-mediated, and lipid raft-mediated endocytosis) or membrane fusion, respectively (O'Donoghue and Krachler, 2016). Several studies have shown that MVs have a multifaceted role both offensively and defensively due to their various components (MacDonald and Kuehn, 2012; Schorey et al., 2015; Guerrero-Mandujano et al., 2017). For instance, many of the vesicles stimulate the activation of the host immune response for the elimination of pathogens, and another mechanism for delivery of the autolysins and virulence factors or cytotoxins is for defending against the digestion and maintaining survival and replication in hosts (Jurkoshek et al., 2016; Li et al., 2018). Therefore, bacterial MVs containing different components bind to certain receptors on macrophages, which can affect the polarization of cells and thus affect the process of inflammation and infection.

### Exosomes of the host in bacterial infections

Extracellular vesicles released by eukaryotes are generally divided into three main populations, including exosomes, microvesicles, and apoptotic bodies (Pfeifhofer-Obermair et al., 2016; Escudé Martinez de Castilla et al., 2021; Tian et al., 2022). In recent decades, exosomes have been found to be important in regulating cell function during bacterial infection. They can be secreted by hematopoietic origin, including macrophages, dendritic cells (DCs), B cells, mastocytes, platelets, and cells of non-hematopoietic origin, such as neurons and epithelial cells (Denzer et al., 2000; Tian et al., 2022; Wang et al., 2022; Zhang et al., 2022). In mammals, exosomes are present in all biofluids, including urine, blood, breast milk, saliva, cerebrospinal fluid, and ascites, and are observed among all kingdoms of life, from bacteria to mammals (Liu et al., 2022). Various databases including EVpedia, Vesiclepedia, and Exocarta have been shown to provide abundant resources for the study of exosomes (Kalra et al., 2012; Kim et al., 2013; Keerthikumar et al., 2016). The studies have demonstrated that exosomes carry proteins, lipids, nucleic acids, and even bacterial components for intercellular communication including the activation or inhibition of recipient cells. The cargoes vary depending on the cell type of origin and physiological/pathological state, which have a broad range of biological functions and participate in multiple physiological and pathological processes such as tumorigenesis, inflammation, immune response modulation, angiogenesis, and tissue repair (Giri et al., 2010; Cheng and Schorey, 2013; Schorey et al., 2015; Palacios et al., 2021). The best-described mechanism for the formation of exosomes is associated with the successive endocytosis of the endosomal system and the internal vesicles of multivesicular bodies (MVBs), which is driven in an endosomal sorting complex required for transport (ESCRT)-dependent or ESCRT-independent manner (Colombo et al., 2014). Certain proteins such as transport and fusion-related proteins, tetraspanins (CD9, CD63, and CD81), and heat shock proteins (HSPs) are contained in exosomes in various levels of expression (Denzer et al., 2000; Schorey et al., 2015), which are commonly used as markers to identify exosomes. Though exosome uptake by target cells depends on the type of recipient cells, phagocytosis and macropinocytosis seem to be involved in this process crucially. Moreover, exosomes can interact with target cells in a ligand-to-receptor manner, including transferrin receptors, TLRs, integrins, and CD94/56 with their individual ligands, respectively (Yáñez-Mó et al., 2015). Taken together, the content of the host-derived exosomes and bacteria-derived MVs can have significant effects on who has the advantage in the battle between the immune system and the pathogen during the infection. Further characterizing the composition and function of EVs will better reveal the biological relevance of these natural nanocarriers and provide new strategies for diagnosis and therapy (Zhang et al., 2021).

## EVs in the regulation of macrophage polarization in microbial infections

Bacteria are major pathogens that develop resistance and cause distinct types of infectious diseases such as tuberculosis, legionnaires' disease, and acute fibrinopurulent pneumonia (Livermore, 2004; Kiszewski et al., 2006; Schwechheimer and Kuehn, 2015; Jung et al., 2016). Independent of the location, microbes have developed many tools to facilitate microbe–microbe, microbe–host, and microbe–environment interactions (Kaparakis et al., 2010; Furuyama and Sircili, 2021; Cui et al., 2022). One strategy is through the classical secretion system types (1~7), which have been widely studied and characterized (El Qaidi et al., 2017; Hui et al., 2018; Grigoryeva et al., 2021; Hardy et al., 2022). The other one depends on MVs, which are considered an alternative and independent secretion system that carries virulence effectors and toxins to regulate the function of recipient cells (Kaparakis et al., 2010; Guerrero-Mandujano et al., 2017; Furuyama and Sircili, 2021). Packaging virulence factors into or onto MV concentrates can increase their stability, allow toxins and virulence factors to be delivered intracellularly, target specific objects at different organelles in host cells to expand or alter their function, and allow them to be transported over long distances (Rüter et al., 2018; Rueter and Bielaszewska, 2020).

### For mycobacterial infections

*Mycobacterium tuberculosis* (Mtb), the major causative agent of TB, is capable of surviving within the phagosomes of host alveolar macrophages and establishes latent infection for the lifetime of the host (O'Garra et al., 2013; Singhania et al., 2018). Previous reports have shown that SecA and Esx protein secretion systems are important for mycobacterial virulence effectors' delivery into the cytosol of the host (Feltcher and Braunstein, 2012; Gröschel et al., 2016). More recently, evolving evidence suggests that Mtb MVs transport various effectors including cell membranes, cell walls, and extracellular proteins into the recipient cells, particularly macrophages, to regulate the host immune response depending on but not limited to nutrient uptake, oxidative stress, envelope stress, and antimicrobial peptides (Jurkoshek et al., 2016; Palacios et al., 2021; Cui et al., 2022).

During the initial infection, Mtb vesicles are recognized and endocytosed by macrophages and then activate M1 polarization, which secretes large amounts of pro-inflammatory mediators, facilitates complement-mediated phagocytosis, and induces type I inflammation to eliminate infection (Anand et al., 2010; Singh et al., 2015; Chiplunkar et al., 2019; Palacios et al., 2021). Surprisingly, exosomes have also been shown to be carriers of some important soluble mediators like cytokines such as IL-1β, IL-6, TNFα, TGFβ, and CCL2/3/4/5 (Yáñez-Mó et al., 2015), which then regulate the function of naïve macrophages. These results indicate besides being released by the cell through the fusion of secretory lysosomes with the plasma membrane, the cytokines are also secreted in exosomes, which are then characterized as pro-inflammatory or anti-inflammatory exosomes, accelerate or suppress the progression of inflammation. It is speculated that cytokines encapsulated in exosomes could preserve more activity than in their soluble form (Schneider et al., 1998). Immune cells infected by Mtb can also release exosomes with or without MVs ingredients, which will influence macrophage polarization and mediate to generate both protective innate and adaptive immune responses against Mtb (Palacios et al., 2021). Mycobacterial antigens including lipoprotein, lipoarabinomannan (LAM), antigenic target protein-6 (ESAT-6), and HspX are reported to be detected in exosomes released from Mtb-infected macrophage J774 cells (Bhatnagar et al., 2007; Giri et al., 2010). Treatment with exosomes carrying these Mtb antigens *in vitro* could promote naïve macrophages to M1 phenotype with the production of pro-inflammatory cytokines (Bhatnagar and Schorey, 2007; Singh et al., 2012; Walters et al., 2013) and enhanced expression of membrane surface markers such as CD40, CD86, CD80, and HLA-DR (Wang et al., 2014, 2015), which indicate that developing immunity against exosomes will increase host resistance to Mtb infection. Taken together, during Mtb infection, in addition, to being released directly by Mtb in soluble form, virulence effectors can also occur in Mtb MVs or exosomes of infected host cells to affect the phenotype of uninfected macrophages.

Research has disclosed that exosomes secreted from other Mtb-infected immune cells such as dendritic cells and neutrophils, can also induce a significant pro-inflammatory response of macrophages, and endothelial cells can be also activated in this process (Marinho et al., 2013; Alvarez-Jiménez et al., 2018; Li et al., 2018). A significant upregulation of genes and proteins known to promote the recruitment and activation of leukocytes is involved in cell adhesion and the inflammatory process through several immune response-related pathways such as TLR2/NF-κB and the type I interferon pathways. This recruitment is supposed to activate a robust innate immune response and helps speed up the removal of pathogens, but sometimes can lead to tissue damage if it lasts too long. DCs, active naïve CD4^+^, and CD8^+^ T cells *in vivo* can be stimulated, indicating a possible application for these exosomes as a TB vaccine for immune defense. Mycobacterial antigen delivery by exosomes to bystander naïve cells to mediate host protection has been suggested to undergo through different mechanisms such as increased phagocytosis (Wang et al., 2015), high production of superoxide (Alvarez-Jiménez et al., 2018), or by phagosome maturation through a non-canonical LC3-associated phagosome pathway in macrophages (Cheng and Schorey, 2019). In summary, these results suggest that MVs are central mechanisms for intercellular communication between bacteria and host cells during infection, and exosome secretion-mediated signal transduction can be beneficial to the host and the pathogen.

**Figure 1:**
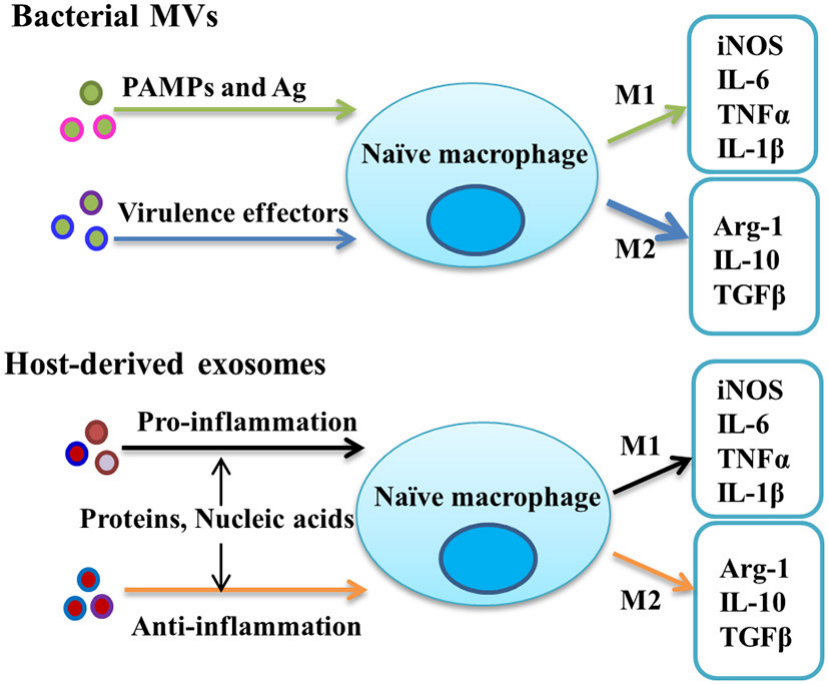
Potential mechanisms for the effect of EVs, including bacterial MVs and host-derived exosomes, on the polarization modulation of uninfected macrophages. Abbreviation: MVs, membrane vesicles.

Although studies suggest that EVs that promote persistent inflammation may be detrimental to the host, it is likely that MV-mediated Mtb–host interactions are more complex and multifactorial, relying on Mtb antigen availability as exosomal content at every step in the process (Wang et al., 2019; Mirzaei et al., 2021). Indeed, early secreted ESAT-6 from Mtb can also directly inhibit the activation of NF-κB and IFN regulatory factors downstream of TLR2 *via* Akt-dependent mechanisms, which suggested that acute mycobacterial infection interferes with M1 polarization (Pathak et al., 2007) and the signal divergence of the same molecule. High and sustained levels of type I interferons from the macrophage and other sources (e.g., T cells or DCs after viral infection) can also be detected at the later stage or latent period of Mtb infection and be harmful to induce the suppressive cytokine IL-10 secretion (McNab et al., 2014; Moreira-Teixeira et al., 2017; Singhania et al., 2018), which may be responsible for the tolerance of low Mtb loads in the host. A possible molecular mechanism is speculated due to the heterogeneous nature of the EVs released by Mtb-infected macrophages, with unknown bacterial molecules possibly present in a vesicle population distinct from the exosome, which then induces different functions on the recipient cells. A comprehensive proteomic analysis confirmed the inference, which identified two distinct, largely nonoverlapping vesicle subsets discovered from Mtb-infected macrophages (Athman et al., 2015; Lee et al., 2015). Besides, the common exosomes with host components, other entirely distinct vesicles are detected to contain a rich source of pathogen-associated molecular patterns (PAMPs) such as bacterial lipoproteins, glycolipids, and LAM. The vesicles harboring these molecules are predicted to suppress the function of macrophage and promote intracellular Mtb survival through activating cell surface and cytosol TLRs for the long term, respectively (Jurkoshek et al., 2016). Consequently, these antigens in MVs transferred to macrophages were released in exosomes which then inhibited IL-2 production and reduced T-cell proliferation to further suppress the adaptive immune response (Jurkoshek et al., 2016; Athman et al., 2017). Although TLR activation is typically important for promoting immunity, prolonged TLR2 signaling during Mtb infection contrary leads to the M2 program and inhibition of Th1 polarization of responding T cells for preventing tissue damage or intracellular survival of bacteria (Singh et al., 2011; Richardson et al., 2015). There is no consensus as to whether these PAMP molecules enter exosomes through MVs or the classical secretory system into MVBs and then are present in the host exosomes or both. MVs carrying metal ions and degradative enzymes could also contribute to nutrient acquisition for bacterial survival. Because metal ions are important for MVs transport during host invasion and transition of the bacterium, which can be confirmed by the presence of different metal ion binding proteins detected by proteomics analysis (Lee et al., 2007). Enzymes found in MVs can also degrade complex biomolecules in the culture medium to make nutrients available (Biller et al., 2014). In general, these results suggest that during acute Mtb infection, Mtb MVs carrying some antigens and virulence effectors can bind to cell surface TLRs and activate macrophages to express the M1 program and release inflammatory exosomes on naïve macrophages to activate them into a pro-inflammatory phenotype, and then participate in the process of eliminating the mycobacterium. On the other hand, prolonged activation by these antigens or other effectors in MVs can also restrain the M1 polarization for their replication and survival (Figure 1). In brief, the EV-modulated polarization of macrophages likely plays a critical role in charging the balance of immunity and immune evasion which is the characteristic of latent Mtb infection.

At present, the mechanism of mycobacterial virulence effectors translocation into EVs and the specificity of this transport remain undefined. SecA2 and Esx-1 protein secretion systems are important for exporting a multitude of specific effectors out of the bacterial cytoplasm and into the cell envelope or extracellular space (Feltcher and Braunstein, 2012; Gröschel et al., 2016), and Esx-1 is required for mycobacterial DNA release into the cytosol of infected cells (Manzanillo et al., 2012). Moreover, Mtb RNA can also be delivered into MVs *via* a SecA2-dependent pathway, as well as through cytosolic and intracellular excesses of infected macrophage, which then induces a more pro-inflammatory response with an increased bacterial killing capacity of the macrophages (Biton et al., 2019; Cheng and Schorey, 2019). These results demonstrate that the SecA2-mediated secretion of bacterial nucleic acids packaged in MVs permits infected macrophages to efficiently detect the presence of viable and virulent Mtb in the cytosol *via* the immune sensory receptor RIG-I. The results not only reveal a novel cytosolic immune sensing strategy for Mtb infection but also suggest that such immune sensing is linked to the recognition of bacterial virulence because MVs transport cargo that serves as virulence factors and/or ligands for cytosolic immune sensory receptors. The mechanism of how these virulence effectors are transported from MVs to exosomes of infected hosts remains inexplicit. There are studies showing that mycobacterial MVs are delivered into the cytosol of macrophages *via* clathrin-mediated endocytosis (Chiplunkar et al., 2019). In addition, Smith et al. have shown that mycobacterial proteins either released by bacteria or endocytosed by macrophages required mono-ubiquitination for trafficking to the exosomes of infected hosts (Smith et al., 2015). Previous studies have shown that EsxB, an ESAT-6-like secreted protein, is encapsulated in Mtb MVs and then delivered into the host cytoplasm to modulate the immune response (Lee et al., 2015), but it remains to be shown whether the secreted protein ESAT-6 is involved in the MVs or only in a soluble state.

### For *Salmonella* infection

*Salmonella enterica* serovar Typhimurium (*S. typhimurium*) is an important cause of gastroenteritis and can invade and survive within macrophages to cause enteric diseases (Gogoi et al., 2019). As a part of the innate immune response, macrophages sense the presence of *Salmonella*-derived PAMPs *via* TLRs. This subsequently leads to an antibacterial response that comprises reactive oxygen species (ROS), reactive nitrogen species (RNS), acidic environment, metal starvation, and antimicrobial peptides (AMPs), which are essential to activate Th1 responses against *Salmonella* to avoid chronic infection. Existing studies have shown that *Salmonella* invasion is mechanistically similar to Mtb infection. MVs released from *Salmonella* are heterogeneous and include mixtures of toxins, transporters, degradative enzymes, and transcriptional regulators which are responsible for the activation of the host immune system or survival of *Salmonella* inside macrophages (Li et al., 1998; Bai et al., 2014). As a defensive strategy, MVs serve as decoys that absorb antimicrobial peptides and neutralize host immune responses. On the other hand, invaded MVs also activate the host's innate immune response (Geddes et al., 2005; Furuyama and Sircili, 2021). It is speculated that macrophages are maintained in a dynamic equilibrium of pro-inflammatory M1 phenotype or anti-inflammatory M2 phenotype depending on the time and dose of bacterial infection and bacterial components, as well as the local environmental stimuli such as different cytokines and downstream signaling pathways (Murray, 2017; Wang, X et al., 2021). In some cases, recruitment, repair, and resolution are rapid (minutes to a few days) for minor damage. It is important to emphasize the time dependence of resolving inflammation, which requires more detailed future studies.

Various exosome subpopulations have been reported to be released from *Salmonella*-infected macrophages with distinct contents and functions. In the early phase of *Salmonella* infection, macrophages can be activated to an M1-like response by producing pro-inflammatory exosomes which then transfer cargo to naïve macrophages to induce their activation (Bhatnagar et al., 2007; Hui et al., 2018). The pro-inflammatory effects are partially attributed to virulence effectors of *Salmonella* such as LPS, *Salmonella* invasion protein A/C (SipA/C), flagellin, and T1SS-secreted agglutinin RTX, which are detected within exosomes. They partially trigger an increased production of pro-inflammatory cytokines in naïve macrophages. However, whether these virulence factors are encapsulated in MVs or delivered into host cytoplasm by T1SS directly as part of the host exosomes or both are undefined. In addition, the following studies suggest that some of the virulence factors encapsulated in MVs can cause violent IL-1β release through the cell pyroptosis pathway to prevent pathogen infection. MVs containing flagellin released from *Salmonella* or *Pseudomonas aeruginosa (PA)* are recognized by TLR5 and endocytosed by macrophages, which trigger inflammasome activation of BMMs through the NLR family CARD domain-containing protein 4 (NLRC4) and caspase-1 pathway and then enhance the production of IL-1β (Yang et al., 2020). However, flagellin-deficient *Salmonella* MVs induced NLRC4-independent non-canonical inflammasome activation and caused a weak interleukin-1β production in an NLR family, pyrin domain containing 3 (NLRP3)-dependent manner, which indicates that OMV-associated flagellin is crucial for *Salmonella* OMV-induced inflammasome response, and NLRC4 is a rapid sensor of bacterial OMV-bound flagellin as a host defense mechanism to promote the removal of bacterial pathogens against infection. Endocytosis partially depends on the transferrin receptor present on the surface of the macrophage, which binds to plasma ferric ion-bound transferring (Gogoi et al., 2019). All these changes in macrophage iron homeostasis are reported to be IFN-γ-mediated. Similar results are found in the treatment of *Salmonella* MVs on chicken macrophages (Cui et al., 2022). Therefore, MVs released from *Salmonella*, like those from Mtb, can carry virulence factors that induce macrophages to polarize toward type M1 and exert the ability to eliminate bacteria. In addition, infected macrophages can also secrete inflammatory exosomes with or without bacterial components to activate uninfected macrophages to type M1 and participate in the clearance of pathogens. The difference is that due to the presence of flagella, *Salmonella* can activate M1-like macrophages more strongly and the signaling pathways are more complex.

On the other hand, in order to survive and replicate in the host, bacterial pathogens are able to manipulate macrophage gene expression to induce the M2 program in order to escape the hostile environment present in M1-polarized macrophages (Owen et al., 2016; Saliba et al., 2016). Studies have shown that *Salmonella* can stimulate an M2 profile of macrophages *via* the production of a key anti-inflammatory cytokine IL-10 through its effector protein SteE (also known as *Salmonella* anti-inflammatory response activator, SarA) to trigger the activation of the host STAT3 and promote intracellular replication and increase virulence (Jaslow et al., 2018; Panagi et al., 2020). Deletion of *Salmonella*-secreted effector K1 (SseK1) can decrease the virulence of *Salmonella in vivo* and *in vitro*. SseK1 can downregulate the inflammation-related cytokines and prevent necroptotic cell death by inhibiting NF-κB signaling to maintain the M2 phenotype, which will be beneficial for *Salmonella* survival (Günster et al., 2017; Lu et al., 2021). Furthermore, iron supplementation is found to increase the intracellular survival of *Salmonella*. The nuclear peroxisome proliferator-activated receptors γ (PPARγ) and PPARδ, *via* their signal transduction, are pivotal in dictating the gene regulation patterns of M2 macrophages. Protein kinase C (PKC) isotypes such as PKCα, PKCβ, PKCδ, and PKCθ, have also been shown to have critical roles in antimicrobial immune responses of the macrophages (Gogoi et al., 2019; Mathieu et al., 2019). The mechanistic details of how SteE and SseK1 drive M2 polarization are lacking entirely, whether they are enclosed in *Salmonella* MVs or just delivered to host cytoplasm through the *Salmonella* SPI-1 and SPI-2 encoded type III secretion system (T3SS) is unknown. Since T3SS is a syringe-like apparatus exploited by some gram-negative bacteria to deliver virulence effectors into infected host cells (Kyrova et al., 2012; Lawrence et al., 2021; Wang, Y et al., 2021), it is possible that virulence factors may be released by the secretory system and then encapsulated in MVs other than in soluble form to maintain their activities.

### For *Escherichia coli* infection

Acute infections with pathogenic *E. coli* cause gastroenteritis, urinary tract infections, acute lung injury (ALI), and sepsis (Mège et al., 2011; Nirujogi et al., 2022). However, *E. coli* strain Nissle 1917 (EcN) can be well colonized in the human intestinal tract and can modulate intestinal homeostasis and microflora balance, which has been developed as a microbial product or dietary supplement to treat intestinal inflammatory diseases such as inflammatory bowel disease (IBD) and infectious diarrhea (Lee et al., 2018; Ramos and Papadakis, 2019; Sanders et al., 2019). Compared to pathogenic *E. coli*, EcN is not pathogenic due to the lack of some defined virulence factor genes in its genome (Grozdanov et al., 2004). Therefore, the genome structures of pathogenic bacteria and probiotics determine the difference between their protective or harmful effects on the host immune system (Grozdanov et al., 2004; Kulp et al., 2015). Consequently, MVs from different strains of *E. coli* will likewise be wrapped with distinct components and perform entirely distinct regulatory roles on cells.

Studies have shown that MV-associated LPS from pathogenic *E. coli* leads to NLRP3-dependent M1-associated cytokine IL-1β secretion *via* LPS delivery into the host cytoplasm and triggers TLR4/TRIF signaling pathway to cause caspase-11-mediated non-canonical inflammasome activation (Santos et al., 2018). Similar results are found in that enterohemorrhagic *E. coli* (EHEC) MVs traffic LPS or heat-labile enterotoxin (LT) into the cytosol of host cells (Vanaja et al., 2016; Rueter and Bielaszewska, 2020). The mechanism further revealed that endotoxins were likely the ligands that mediated the binding of MVs to lipid rafts of host cells, thus leading to the uptake of MVs. Based on high sequence homology to SseK1, a unique T3SS effector of *Salmonella, E. coli* effector NleB1 can block TNF-mediated NF-κB pathway activation to inhibit antibacterial and inflammatory host responses (El Qaidi et al., 2017). Whether NleB1 is packaged in OMV remains undefined. Several articles have identified that bacterial MVs enter epithelial cells *via* NOD receptor-dependent NF-κB pathways or lipid rafts and caveolin-dependent endocytosis (Kaparakis et al., 2010; Cañas et al., 2018). For macrophages, the uptake of *E. coli* MVs may also be through random phagocytosis, classic endocytosis, or specific pathways, and the detailed mechanisms need further investigation.

As probiotics, EcN MVs can recapitulate the anti-inflammatory properties of EcN by modulating cytokine expression and production from various cells and tissues in different manners. In peripheral blood mononuclear cells (PBMCs) and intestinal epithelial cells, a mixed secretion of M1-associated pro-inflammatory cytokines IL-6, IL-8, and TNF-α with M2-associated anti-inflammatory cytokines IL-10 can be triggered (Fábrega et al., 2016; Cañas et al., 2018). In other studies performed *in vivo* and *in vitro*, EcN MVs have been shown to be effective in enhancing the antibacterial activity of macrophages, which can regulate the adaptive immune response to host defense (Hu et al., 2020). The reasonable causes are that the upregulation of pro-inflammatory cytokines activated by EcN MVs is probably due to LPS or other PRR ligands, while some other unidentified vesicular components may induce the activation of M2-associated cytokines (Kulp et al., 2015). These findings again emphasized that M1- and M2-polarized phenotypes could be provoked by bacterial MVs with different components at the same time and they will interact with each other in the presence of *E. coli* anti-inflammatory response.

Other than MVs, exosomal shuttles from *E. coli*-infected cells can transfer cargo from cell to cell and affect the function of recipient cells. The pathogenic adherent-invasive *E. coli* (AIEC), which abnormally colonizes the intestinal mucosa of patients with CD, is able to adhere to and invade intestinal epithelial cells (IECs), survive and replicate within macrophages (Lapaquette et al., 2012; Nguyen et al., 2014; Mitsuhashi et al., 2016; Ramos and Papadakis, 2019). Studies have reported that pro-inflammatory exosomes are enhanced in the patient's lumen after AIEC infection. The secretion of exosomes by human IECs and THP-1 macrophages *in vitro* is promoted as well, which are in turn entrapped by naïve THP-1, leading to increased pro-inflammatory response with the elevated secretion of M1-associated cytokines through pathways involving NF-κB, p38 MAPK, c-Jun N-terminal kinase, and impaired clearance of intracellular AIEC in exosome-receiving cells (Carrière et al., 2016; Larabi et al., 2020). Exosomes released by AIEC-infected IECs also inhibited autophagy-mediated clearance of uninfected AIEC due to increased levels of miRNA-30c and miRNA-130a packaged in exosomes through inhibiting ATG5 and ATG16L expression, thus favoring AIEC intracellular replication within IECs (Larabi et al., 2020). Whether such a similar signaling pathway also occurs in *E. coli* intracellular survival and replication of macrophages requires further verification.

Bacterial effectors can act on recipient cells by being encapsulated in MVs or transported through undefined mechanisms into the infected host exosomes. Exosomes released from Shiga toxin 2a (Stx2a)-treated human THP-1 macrophages contain Stx2a, modulate inflammatory responses, and induce cell death in human renal cortical epithelial cells expressing the toxin receptor globotriaosylceramide (Gb3) (Lee et al., 2020). The high expression of pro-inflammatory cytokines IL-6, TNFα, IL-1β, and IL-8 indicates an activated M1-polarized phenotype after infection. All pro-inflammatory cytokines are packaged randomly into diverse Stx2-associated exosomes, but only exo-mRNA levels of IL-1β and IL-8 are higher than those in uninfected THP-1 cells, which may be the reason for the exacerbated localized inflammation and death of recipient cells in Stx-mediated renal injury. Regardless of nucleotide and protein cargos packaged in exosomes, lipid mediators can also control the initiation and resolution of acute lung inflammation (Ott et al., 2011; Robb et al., 2016). A possible mechanism has been revealed recently, increased release of exosomes from alveolar macrophages which carried a diverse array of lipid mediators derived from ω-3 and ω-6 polyunsaturated fatty acids (PUFAs) metabolite profile in part depend on the inflammatory status of the lung macrophages and their interaction with other lung cells in *E.coli* LPS-activated ALI (Nirujogi et al., 2022). However, the processes of lipid mediator synthesis and transportation are much more complicated, which will need to be further delineated in the future.

### For other bacterial infections

In addition to the bacteria mentioned above, there are a number of other bacteria that affect the inflammatory process by regulating the proportion of M1/M2 macrophages by EVs after infection, which are shown in Table 1.

**Table 1:** Molecules of EVs and their roles in modulation of macrophage polarization.

**Molecule in EVs**	**Origin of vesicles**	**Effect on macrophages**	**References**
LprG, LpqH, Phos1, LAM	*Mycobacterium tuberculosis* (Mtb)	M1	Athman et al. (2015), Jurkoshek et al. (2016)
Lipopeptides	Mtb-infected dendritic cells	M1	Marinho et al. (2013)
	Mtb*-*infected neutrophils	M1	Alvarez-Jiménez et al. (2018)
Mtb RNA	Mtb-infected macrophages	M1	Singh et al. (2015), Cheng and Schorey (2019)
LPS GPLs, ESAT-6, Hsp X, LAM, lipoprotein	*Salmonella*-infected macrophges, Mtb-infected macrophages	M1	Bhatnagar and Schorey (2007), Bhatnagar et al. (2007), Giri et al. (2010)
LAM	Mtb	M2	Jurkoshek et al. (2016)
flagellin with LPS	*Salmonella*, *Pseudomonas Aeruginosa (PA)*	M1, inflammasome	Yang et al. (2020), Cui et al. (2022), Liu et al. (2022)
LPS, flagellin SipA/C, RTX	*Salmonella-*infected macrophages	M1	Hui et al. (2018)
LPS	*E. coli* *E. coli-*infected macrophages	M1, inflammasome	Santos et al. (2018), Nirujogi et al. (2022)
	*E. coli*-infected macrophages	M1	Carrière et al. (2016)
Stx2a, mRNAs of IL-1β and IL-8	Stx2a of *E.coli*-treated macrophages	M1	Lee et al. (2020)
microbial molecules	*E. coli Nissle 1917*	M1, M2	Alvarez et al. (2019), Hu et al. (2020)
LPS, LOS	*Haemophilus*, *Influenza*, *Moraxella catarrhalis*, *PA*	M1	Volgers et al. (2017a)
	*Legionella pneumophila*	M1 M2	Burstein et al. (2016), Jung et al. (2016)
OprF	*PA*	M2	Armstrong et al. (2020), Moussouni et al. (2021)
LPS, OprC	*PA*	M1	Gao et al. (2020), Jia et al. (2020)
Epi_2D, Pro_mqo, Pro_ca	*Helicobacter pylori*	M2	Ahmed et al. (2021)
OipA	*Helicobacter pylori*	M1	Soudi et al. (2020)
OmpU	*Vibrio cholerae*	M1	Khan et al. (2015)
	*Moraxella catarrhalis*	M1	Volgers et al. (2017b)
LPS	*Acinetobacter baumannii*	M1	Li et al. (2015), Chiu et al. (2020)
OmpA	*Acinetobacter baumannii*	M1 Cell death	Skerniškyte et al. (2021), Tiku et al. (2021)
RNAs	*Aggregatibacter actinomycetemcomitans*	M1	Han et al. (2019)
Opa	*Neisseria gonorrhoeae*	M1	Makepeace et al. (2001)
Sphingolipids, Arg- and Lys-gingipain	*Porphyromonas gingivalis*	Inhibition of M1	Rocha et al. (2021), Castillo et al. (2022)
	*P. gingivalis*	M1, M2 inflammasome	Fleetwood et al. (2017)
	*Treponema denticola, Tannerella forsythia*, *P. gingivalis*	M1, inflammasome M1 M1, M2	Cecil et al. (2017)
Tube-shaped vesicles	*Francisella novicida*	M1	McCaig et al. (2013)
	*Fusobacterium nucleatum*	M1	Chen et al. (2022)
PorB	*Neisseria gonorrhoeae*	Cell apoptosis	Deo et al. (2018)

LprG, Lipoproteins G; LAM, Lipoarabinomannan; LOS, lipooligosaccharide; CFB, complement factor B; SipA, *Salmonella* invasion protein A; MAPK, mitogen-activated protein kinase; ESAT-6, antigenic target protein-6; GPLs, Glycopeptidolipids; OMP, outer membrane protein; Stx, Shiga toxin; Gb_3_, globotriaosylceramide; NLRP3, NLR family, pyrin domain containing 3; LC3/MAP1LC3, microtubule-associated protein 1 light chain 3; ASC, apoptosis-associated speck-like protein containing CARD; TRIF/TIRAP, toll-interleukin 1 receptor (TIR) domain-containing adaptor protein; MyD88, myeloid differentiation primary response gene 88; AIM2, absent in melanoma 2; Epi_2D, Epimerase_2 domain-containing protein; LOS, lipooligosaccharide; IRAK1, interleukin1-receptor associated kinase 1; Opa, opacity; PorB, pro-apoptotic VDAC-like porin.

Besides virulence effectors, there are many other cofactors involved in MVs generation. TseF secreted by H3-T6SS of *PA* is incorporated into MVs by directly interacting with the iron-binding *Pseudomona*s quinolone signal (PQS), which suggests a possible approach of general secretion system effectors encapsulated into MVs and an important role of the quorum sensing (QS) system in OMV formation (Lin et al., 2017). In addition, sphingomyelin mutation and inhibition of Arg- and lys-gingipain in MVs of *Porphyromonas gingivalis* can significantly increase the secretion of inflammatory cytokines and chemokines by M1-type macrophages (Rocha et al., 2021; Castillo et al., 2022), possibly suggesting that natural MVs with normal sphingomyelin and Arg- and lys-gingipain activity may inhibit the M1-type polarization of macrophages during bacterial infection and prevent macrophages from playing a scavenging role. These results indicate that MVs secreted by different strains of bacteria or by different treatment methods contain distinct components that have greatly different effects on macrophage function. As an opportunistic nosocomial pathogen, *Acinetobacter baumannii (A. baumannii)* can activate pro-inflammatory macrophages reaction, evade neutrophil chemotaxis, and cause cell death through the cytotoxic Outer membrane protein A (OmpA) (Knapp et al., 2006; Bhuiyan et al., 2016; Skerniškyte et al., 2021; Tiku et al., 2021), which is reported to be translocated into the mitochondria and nucleus of target cells to induce fragmentation through being packaged in MVs (Choi et al., 2008; Bhuiyan et al., 2016; Tiku et al., 2021). The results indicate that these effectors are not only limited to inducing the production of inflammatory factors but also have toxic effects on the organelles of the host. Moreover, MVs isolated from two clinical *A. baumannii* strains exhibit different toxicity and proteome characteristics, which suggest that the multidrug-resistant strain containing more virulence factors might produce abundant MVs facilitating the worse outcome (Li et al., 2015). The abundance of proteins correlated with redox and iron metabolism in *A. baumannii* for infection and survival is identified, and others are enriched in the pathways such as platelet activation and signaling, high-density lipoprotein (HDL) remodeling, heme homeostasis, and apoptosis, which indicate the complicated pathogen–host interaction (Kho et al., 2022). Further projects should classify the virulence effectors contained in MVs of various bacteria and reveal the mechanisms of MVs transportation into the host.

**Figure 2:**
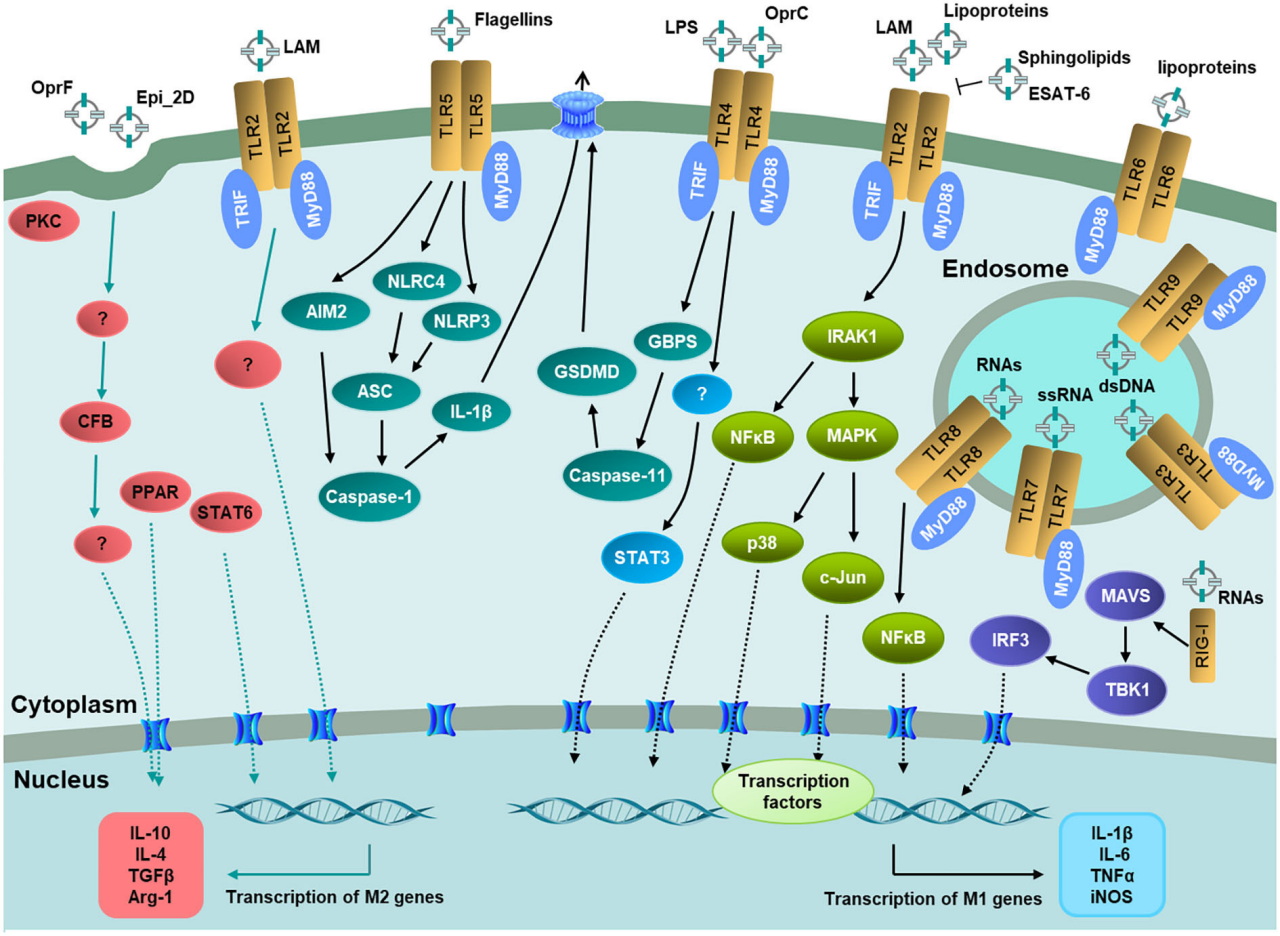
Recognition of EV-associated molecular patterns by host immune receptors and signaling pathways on macrophage polarization. TLR2, TLR4, TLR5, and TLR6 located at the host cell membrane. TLR3, TLR7, TLR88, and TLR9 located at the endosomal membranes. RIG-I located in the cytosol to recognize bacterial RNAs in EVs. The downstream signaling pathways lead to the activation of transcription factors including NF-κB, STAT3, PPAR, and STAT6, respectively, and then the transcription of pro-inflammatory or anti-inflammatory genes. Adaptor molecules: MyD88 and TRIF.

The mechanisms of EVs on macrophage polarization are far more complicated. As important PRRs on macrophages, some TLRs reside at the host cell membrane and recognize bacterial LPS, lipoproteins, and flagellins in MVs or soluble form, while others locate at the endosomal membranes bind MV-associated nucleic acids, respectively (Lim and Staudt, 2013; Liu et al., 2022). The transducers of NF-κB and MAPK are conventional signaling pathways to induce pro-inflammatory cytokines released after EVs bind to individual receptors (Figure 2). For IL-1β, there is another secretion approach by caspase-1-dependent NLR4 activation pathway, which generally causes pyroptosis to prevent bacterial replication and diffusion (Lee et al., 2020; Rueter and Bielaszewska, 2020; Chen et al., 2022). These results provide a therapeutic basis for preventing the excessive release of inflammatory factors. Nevertheless, most of the published reports mainly focused on the mechanism of M1 polarization induced by EVs in macrophages. Future studies may focus on the mechanism of EVs on M2 polarization, to provide a reference for the prevention and treatment of chronic infection.

## Conclusion

During a bacterial infection, the host immune system is exposed to intact bacterial and microbial components, and both are key compounds to control the infection program. Extensive studies have shown that bacterial invasion can regulate the polarization of macrophages by secreting virulence factors through the general secretion system, thus, affecting the development of inflammation. Various reviews have demonstrated the mechanism of macrophage polarization regulation at different stages of bacterial infection, but none focused on the effect of bacteria-derived MVs and host-derived exosomes on macrophage polarization in inflammation. Emerging studies have demonstrated that bacteria can secrete EVs like MVs containing virulence factors to modulate the polarization of macrophages. Host cells infected by bacteria can similarly secrete EVs such as exosomes bearing various proteins or nucleic acids to induce the polarization of uninfected and infected macrophages. Here, we outline the current state of the articles and summarize the effects of EVs from both bacteria and hosts on macrophage polarization in order to better understand the mechanisms underlying the development of disease caused by bacterial infection.

Extracellular vesicle-mediated polarization of macrophages may promote or inhibit the development of infection. Many bacterial productions are involved in MVs and then transported into recipient cells of the same species, other bacterial species, or eukaryotic cells to modulate the cellular processes. The bioactive component of MVs is different for each species and determines whether MV secretion promotes bacterial virulence, host immunity, or both. During the bacterial infection, immune cells like macrophages can uptake the bacterial MVs through different TLR pathways or endocytosis and then activate to release pro-inflammatory or anti-inflammatory cytokines in soluble form or in exosomes with or without bacterial effectors, which then affect the functions of other naïve recipient cells nearby or further apart. In the process of anti-infection, macrophages keep the balance of inflammatory response through the transformation of phenotype. The pro-inflammatory M1 phenotype is prominent during the initial stage of infection, while the anti-inflammatory M2 phenotype dominates during the late stage of infection to prevent excessive inflammatory reactions. There are so many intermediate stages that suggest dynamic equilibrium depending on the course of infection and the dose of bacteria. Some of the most important steps that must be taken in this area are a comprehensive comparison of various subtypes of EVs, such as MVs from bacteria and exosomes from host cells. Different EVs may be effectively distinguished based on their size, density, morphology, and specific surface marker proteins. This is crucial in determining which EVs should be targeted for any therapeutic approach. Moreover, in accordance with the specific biomarkers of different origins of exosomes isolated from bodily fluids such as blood, urine, and saliva, the infection-causing pathogens may be determined. The number of exosomes and their composition can also tell the infection progression, which could provide a targeted treatment program. However, the complex effect of EV secretion on disease pathogenesis must be assessed on a case-by-case basis for each pathogen. In addition, it remains to be further confirmed how these virulence factors enter the exosome of the host cell, whether they are encapsulated in the exosome after being released from the bacterial MVs or secreted into the extracellular cell through the entire vesicle. The exosomes secreted by macrophages after bacterial infection contain not only proteins and mRNA of the host but also miRNAs or lncRNAs (long non-coding RNAs), which should also be taken into account for regulating the gene expression of recipient cells. Defining and classifying the composition and effects of these EVs in the regulation of macrophage polarization, and how they disseminate during infection, is essential to our understanding of the pathogenesis of human diseases and how our immune system responds to the infection.

## Author contributions

MQ wrote and edited the manuscript. HZ revised the manuscript. XZ reviewed and edited the manuscript. All authors read and approved the final version.
